# Supplementary material for: Creatine Monohydrate and Conjugated Linoleic Acid Improve Strength and Body Composition Following Resistance Exercise in Older Adults
Source: PLoS One. 2007 Oct 3;2(10):e991. doi: 10.1371/journal.pone.0000991 (PMC1994592; doi:10.1371/journal.pone.0000991)
Supplement: Protocol S1 — (0.14 MB DOC) [file pone.0000991.s001.doc]

**Summary of Original Grant Proposal to CIHR and Original Ethics Application**

**Tarnopolsky, M.A., et al.**

**Original Title of Proposal: The effect of resistance exercise training and nutritional supplementation on strength, function and muscle mass in older adult men and women.**

**Source of Funding: Canadian Institute for Health Research (Institute of Aging), Date of Funding Decision = April, 2003 (Application # 108073).**

**Summary of Research Proposal. Tarnopolsky, MA (33248)**

***Introduction.***

Aging is characterized by a loss of muscle (sarcopenia) and bone (osteopenia) mass. Strength exercise has been shown to increase muscle mass/strength and bone mass in older adults. We and others have found that short-term creatine monohydrate (CrM) supplementation can enhance the benefits from strength exercise training in older adults. We have recently found that conjugated linoleic acid (CLA, an essential fatty acid) increased bone mass in rats even when treated with corticosteroids. To date, the effects of CLA on muscle and bone, +/- CrM, has not been evaluated in sedentary or strength trained older adults. Together, CLA and CrM supplementation should complement each other’s beneficial effects on bone and muscle mass, respectively. The benefits of the CLA/CrM combination should be magnified with the additional stimulus of strength exercise training. Given the increase in the proportion of adults over the age of 65y, data from the current proposal could lead to the development of safe and relatively inexpensive interventions for sarcopenia and osteopenia.

## *Objectives.*

## To determine the pattern of gene expression in skeletal muscle in older adults in response to strength exercise training and nutritional interventions with CLA and creatine.

- To asses whether CLA and creatine monohydrate work independently or in combination with strength exercise to increase bone mass/density and muscle mass and function.
- To determine whether the benefits from strength exercise are related to a reduction in oxidative stress and mitochondrial DNA deletions and whether strength exercise training enhances mitochondrial function of the skeletal muscle and at the whole body level.

***Hypotheses.***

Resistance exercise training will result in:

- ↑ strength, fat-free mass, aerobic capacity, bone mineral content, muscle fiber area, mitochondrial enzyme activity and a reduction in mitochondrial DNA deletions.
- ↓ in the “stress pattern” from the micro-array data along with an increase in expression of mRNA species associated with the electron transport chain of the mitochondria.

CrM + CLA will result in the following adaptations that will be enhanced with strength exercise:

- ↑ strength and functional tasks, muscle total and phosphocreatine, fat-free mass and bone mineral content and osteocalcin (bone formation).
- ↓ oxidative stress markers and N-telopeptides (bone breakdown).

***Research Plan.***

*1. Subjects.* N = 80 healthy males and females 65 - 85 y. *2. Design/Intervention.* Randomized, double-blind study with equal allocation to 4 treatment groups: 1. Resistance training (RT) (8 months, 3d⋅wk^-1^) and placebo (PL) – Group = RT + PL; 2. RT as in # 1 + CrM (5 g⋅d^-1^) and CLA (3 g⋅d^-1^) supplementation– Group = RT+SUPP; 3. Sedentary (8 months of home stretching (SED)) and placebo (PL) = SED; 4. SED as in # 3 + SUPP as in # 2 – Group = SED+SUPP. *3. Major outcome variables.* A. Function – muscle strength (isometric and dynamic), aerobic capacity, functional tasks; B. Anthropometry – DEXA for fat-free mass, bone mineral content and density; C. Muscle – Histology – ATPase for fiber area and type, cytochrome oxidase (COX) negative fibers, COX sub-unit II and IV and 8-OH-2deoxyguanosine by IHC; Biochemistry – electron transport activity, creatine, ATP, phosphocreatine, protein carbonyls and anti-oxidant enzymes; Molecular biology – mitochondrial DNA deletions, gene expression pattern by micro-chip technology; D. Blood – CK activity, liver and renal function tests, bone turnover markers (osteocalcin and N-telopeptide); E. Urine - 8-OH-2deoxyguanosine/creatinine.

**APPLICATION FOR REVIEW BY RESEARCH ETHICS BOARD**

**For use by investigators performing research to be reviewed by the Research Ethics Boards (REBs) of:**

**St. Joseph’s Healthcare Hamilton; Hamilton Health Sciences/McMaster University, Faculty of Health Sciences; and other affiliated institutions.**

*This form is available in MS WORD & WP formats and can be downloaded at:* <http://www.fhs.mcmaster.ca/csd/forms/downloads.htm>

## *Complete the application in NO smaller than 12 point font; handwritten submissions are NOT acceptable*

***(REVISED August 2002*)**

***Please refer to the Tri-Council Policy Statement on Ethical Conduct for Research Involving Human (1998) prior to completion of this form -*** [***http://www.nserc.ca/programs/ethics/english/policy.htm***](http://www.nserc.ca/programs/ethics/english/policy.htm)

Is this a student project? [ ] Yes [X ] No Project # ____________________

(will be assigned by REB)

**SECTION A – GENERAL INFORMATION**

1. (a) Title of Project: The effect of resistance exercise training and nutritional supplementation on strength, function and muscle mass in older adult men and women.

(b) List up to 5 keywords that describe this project: aging/dietary supplements/bone health/exercise training

University

1. Name(s) & Degree(s) Title(s) or Clinical Hospital

of Investigator(s) Position(s) Program Affiliation Phone # Email

(a) Locally Responsible Investigator (must have an appointment at the institution where the REB application is being

submitted for review)

Mark Tarnopolsky, MD, PhD. Assoc. professor Neuro/Rehab HHSC 76593 tarnopol@mcmaster.ca

(b) Principal Investigator (if different from (a) above)

(c) Co-investigator(s)

Timothy Doherty, MD, PhD Assist. Prof. Rehabilitation SJH- London Tim.Doherty@sjhc.london.on.ca

(d) Study coordinator

To be hired for the study – minimum of MSc

1. Proposed Date: (a) of commencement: Sept. 2003 (b) of completion: Dec. 2004
2. Indicate location(s) where research will be conducted:

McMaster University: [X] Specify site(s): Training in the Ivor Wynne Center at the Center for Activity and Aging.

Hamilton Health Sciences: [X ] Specify site(s): Testing in the 4U and 2F area (MUMC).

Hamilton Regional Cancer Centre: [ ] Other: [X ] Specify site(s): London Ontario - Centre for Activity and Ageing.

1. (a) Is this a multi-centered study? [X ] YES [ ] NO

(b) Will the application be reviewed by other Research Ethics Boards (REB)? [ X ] YES [ ] NO

If YES, please attach any REB approvals from other jurisdictions. [ ] Attached [ X ] To Follow

- 1. Has the study been denied approval by any other REB? [ ] YES [X ] NO

If YES, please attach REB letter. – simultaneous review

1. Status of Funding: Applied [ ] Date submitted:

Funded [X] Date of decision: Jan, 2003

None [ ] Date:

In-Kind support only (drugs, equip, devices) [ ] Specify contribution:

Name(s) of Research Sponsor/Funding Agency/Industry Partner:

CIHR

Total Budget: $ 189,288 Local Budget: $ Attach copy of Budget Summary – Appendix 1

Where will funds be administered? HHS: [X ] McMaster: [ ] HRCC: [ ]

St. Joseph’s: [ ] Other: [ ] Specify:

1. Conflict of Interest:

Does the principal investigator(s) or any co-investigators involved in this research study:

1. Function as an advisor, employee, officer, director or consultant for the sponsor? [ ] YES [X] NO
2. Have direct or indirect financial interest in the drug, device or technology employed

(including patents or stocks) in this research study? [ ] YES [X] NO

1. Receive an honorarium or other benefits from the sponsor (apart from fees

for service)? [ ] YES [X ] NO

- 1. If the answer is YES to any of the above, please describe and explain how that conflict

is being managed to ensure that participant rights and welfare are not affected. [ ] Attached

1. Sponsor Agreement:

*(Note: Agreements must be reviewed and signed by authorized institutional officials.)*

Will there be a signed contract/agreement with the sponsor related to this study? [ ] YES [ ] NO

If YES, will it in any way limit your access to the research data, or limit your right to

publish the study results? [ ] YES [ ] NO

If YES, please explain.

1. New Investigational Drug or Device, and Off-label Use:

Is this a clinical study involving a new investigational drug(s) or device, or a drug or

device used for an indication outside the parameters of the approved Health Canada

Notice of Compliance (NOC) or Drug Identification Number (DIN) application or Medical

Device Licence, as applicable? [ ] YES [ X] NO

If YES, has the Sponsor or the Institution/Investigator initiating the study filed a Clinical

Trial application or Medical Device Licence Application with Health Canada? [ ] YES [ ] NO

If YES, attach a copy of the Health Canada “No Objection” letter.

*(Note: Study may NOT commence without Health Canada approval.)*

10. (a) Does this study involve an infectious agent? [ ] YES [ X] NO

If YES, attach approval letter from Infection Control.

(b) Does this study involve a biosafety hazard? [ ] YES [ X] NO

If YES, attach approval letter from the Biosafety Committee.

(c) Does this study include genetic testing or storage of tissues/samples? [ ] YES [X] NO

If YES, attach a separate consent form addressing the special concerns

related to genetic testing or storage of tissues/samples. *(Note: See Informed Consent Checklist attached.)*

**SECTION B – DESCRIPTION OF PROPOSED RESEARCH**

**PART I: BRIEF SUMMARY (4 – 5 lines) OF PROPOSED RESEARCH WRITTEN IN SIMPLE ENGLISH APPROPRIATE FOR LAY REVIEWERS (Do not use jargon or medical short forms). USE THE FOLLOWING HEADINGS;**

**(i) Main Research Question(s)** – Do the dietary supplements creatine monohydrate and conjugated linoleic acid enhance the benefits of strength exercise training in older adults and do they increase muscle function, mass and bone density on their own?

**(ii) Why is this research important? –** Muscle weakness and bone loss are major causes of disability in older adults and strategies that enhance muscle and bone mass should have significant beneficial effects on health and mobility of older adults.

**(iii) What is being studied? –** Muscle strength, aerobic fitness, bone mass, functional capacity will be determined before and after one year of supervised weight training in older adults as compared to a cohort of adults who do not exercise. Within each group, half will be taking the dietary supplements, creatine monohydrate and conjugated linoleic acid.

**PART II: (THIS SECTION MUST BE COMPLETED EVEN IF PROTOCOL IS ATTACHED)**

1. Describe the rationale and purpose of the study (250 words):

Aging is characterized by a progressive loss of muscle mass, termed, sarcopenia. Decreased strength ultimately results in an increased occurrence of falls, which are a primary cause of morbidity. Resistance exercise can partially reverse many of these changes, however, long-term compliance limits the broad application of exercise as a counter-measure for sarcopenia. We have recently shown that a dietary substance, creatine monohydrate (CrM) enhanced several of the gains following four months of exercise training in older adults (i.e., fat-free mass, knee extension strength)(Brose, et al.,  *J Gerontol A Biol Sci Med Sci.*, 58:B11-19, 2003). Another compound, conjugated linoleic acid (CLA), has been found to increase muscle mass and decrease fat mass in humans. Our purpose is to study the effects of these dietary compounds in combination with and without strength exercise training in older adults to determine the effects on muscle mass, bone mass and muscle function after one year of intervention.

1. Sample size *(Indicate how the sample size was determined and # of participants involved locally and in total for multi-centered studies)*:

Using the data from our pilot study looking at CrM and strength exercise in older adults (Brose, et al., *J Gerontol A Biol Sci Med Sci.*, 58:B11-9, 2003) we derived sample size estimates for two of the primary outcome variables:

- - 1. FFM (fat-free mass): The change in FFM for CrM after training was 1.92 kg (SD = 0.98) and for PL was 0.53 kg (SD = 0.38). The delta between the groups is 1.39 with a mean SD of 0.68. Hence using a conservative 2 tailed test, and α = 0.05 and β 0.20, the sample size comparing 2 independent groups is: n/group = 2[(Z_α_ + Z_β_) SD/delta]^2^ = 2[(1.28 + 1.96)0.68/1.39]^2^ = 10.
    2. Isometric knee extension strength: The change in strength for CrM after training was 52 N⋅m (SD = 38) and for PL was 24 N⋅m (SD = 13). The delta between the groups is 28 with a mean SD of 25.5. Hence using a conservative 2 tailed test, and α = 0.05 and β 0.20, the sample size comparing 2 independent groups is: n/group = 2[(Z_α_ + Z_β_) SD/delta]^2^ = 2[(1.28 + 1.96)20/28]^2^ = 21.
  1. The comparison between the effect of strength training and the sedentary group for almost every variable of interest resulted in sample size estimates of less than 10/group.
  2. In our pilot study we had a drop-out rate of 7 %, and if we assume a drop-out rate of twice this (~ 6/group), we should retain 34 people at each of the two centers with 34 in RT and 34 on the combined supplement.

1. Design/Methodology:

Participants who are deemed eligible to participate will be randomly allocated into one of four groups. The allocation will be based upon an expected equal recruitment at the London (total = 40) and Hamilton (total = 40) sites and there will be 10 independent blocks of 4 randomly distributed interventions at each site (20 allocated to strength exercise (10 = + supplement; 10 = no supplement) and 20 allocated to no exercise (10 = + supplement; 10 = no supplement)) with an equal distribution:

1. Resistance training (RT) (12 months, 3d⋅wk^-1^) and placebo (PL) – Group = RT + PL.

2. RT as in # 1 + CrM (5 g⋅d^-1^) and CLA (3 g⋅d^-1^) supplementation (SUPP) – Group = RT+SUPP.

3. Sedentary (12 months of home stretching (SED)) and placebo (PL) = SED.

4. SED as in # 3 + SUPP as in # 2 – Group = SED+SUPP.

1. Setting (e.g. outpatient, ICU, community):

The participants will be healthy ambulatory community dwelling men and women over the age of 65 y.

a. Exercise testing – Rm 4U18 and 2F MUMC (Neuromuscular research testing area – Dr. Tarnopolsky).

b. Exercise training – Tue and Thur. – Ivor Wynne Center exercise center.

1. (a) Brief description of participants:

Men and women over the age of 65 y and less than 85 y who are living independently in the community with no contra-indications to exercise testing or training will be recruited.

(b) Are there any age, ethnicity, language, gender or race-related inclusion or exclusion criteria? [ ] YES [ X ] NO

If YES, please justify:

We plan to recruit equal numbers of men and women.

1. Study interventions:

a) Exercise training (50 % of recruits; other 50 % will continue with their usual routine) – participants will perform supervised strength/resistance exercise three times a week (Tue/Thur) for **12 months** using a machine based whole body exercise program that we have shown previously to be safe and effective (Brose, et al.,  *J Gerontol A Biol Sci Med Sci.*, 58:B11-9, 2003). Subjects will also perform a home based exercise routine on Saturday with rubber bands and resistance exercises not requiring weights (i.e., sit-ups).

b) Nutritional supplement (50 % of recruits; the other 50 % will consume a placebo (nested within the exercise and sedentary groups) – the supplement will consist of 3 g/d of conjugated linoleic acid and 5 g/d of creatine monohydrate for **12 months**.

1. Primary outcome(s) (***“What”*** will the study measure?):

The following measurements will be completed before (pre), at 6 months, and again at the end (+12 months) of the study:

a) Muscle and bone mass.

b) Functional capacity.

c) Knee extension strength.

d) Strength with weight training machines.

e) Muscle fiber area and creatine content.

f) Muscle oxidative capacity.

g) Aerobic power (oxygen consumption).

1. Measurements (***“How”*** will the study measure it?) and plan for data analysis:

a) Muscle and bone mass – DEXA scanning.

b) Functional capacity – stair climb, get up and go and walking test.

c) Knee extension strength – isometric dynamometery.

d) Strength with weight training machines – peak strength in 8 muscle actions.

e) Muscle fiber area and creatine content (muscle biopsy pre and post only) – ATPase staining and enzymatic biochemistry.

f) Muscle oxidative capacity (muscle biopsy pre and post only) – electron transport enzyme activity - biochemistry.

g) Aerobic power (oxygen consumption) – VO2max test + 12 lead ECG on cycle ergometer.

**SECTION C – DESCRIPTION OF THE RISKS AND BENEFITS OF THE PROPOSED RESEARCH**

1. What are the proposed benefits to the participants, the scientific community or society that would justify asking individuals to participate?

Society - Sarcopenia is indirectly responsible for many age related morbidities such as falls and bone fractures, muscle weakness a loss of functional independence. Resistance exercise is an effective countermeasure to sarcopenia, however, there are issues of poor long-term compliance. Substances that can enhance the effects or resistance exercise or work in isolation would be of immense benefit to older adults with sarcopenia and also for healthy older adults (such as in the current proposal) to prevent sarcopenia.

Individual – Each participant will have an exercise stress test, a nutritional assessment and body composition analysis (including bone density). These test results are of interest to many people and may identify aspects of health that need preventative attention such as low bone mineral density. 50 % of the participants will have supervised exercise training for one year (~ 150 – 400 dollar benefit, depending on the health club). All participants will be taught a stretching program. At the end of the study, any of the people in the non-exercising program will be offered a two session supervised instruction period to help them get starting in and exercise program if they desire.

Scientific Community – There will be a number of outcome variables that will answer important questions about aspects of muscle adaptation to exercise. An example is the hypothesis that satellite cells may function as stem cells and bring non-mutated mitochondrial DNA into the muscle fibers that can alter the ratio of mutated mitochondrial DNA (deletions) toward a younger profile.

1. Specify the risks to participants involved in this study [e.g. physical risks (pain, discomfort, injury, side effects), psychological risks (participants feel demeaned, worried, upset, embarrassed), social or economic risks (invasion of privacy or breach of confidentiality).

- Creatine has been used by millions of athletes throughout the world for over 5 years. There is one report of an individual with pre-existing renal disease who showed an apparent worsening of kidney function while supplementing and another report of interstitial nephritis in a young man taking twice the recommended maximal dose for 1 month. Because of these case reports we will be conservative and exclude those with known or the potential for (i.e., diabetes) kidney disease. There have been 2 longitudinal studies using creatine for > 1 month and neither has shown any significant adverse effects. All blinded trials that have measured renal function have not shown any deterioration. There have been some anecdotal reports of stomach cramps, however, this appears to be related to the consumption of un-dissolved powder in a minority of subjects. There have been 3 prospective trials in older adults (> 65y) with exercise training and creatine interventions with no reported side effects. We found no evidence of subjective side effects nor alterations in renal and hepatic function in our 4 month trial in older adults (Brose, et al.,  *J Gerontol A Biol Sci Med Sci.*, 58:B11-9, 2003).
- CLA. There have been no reports of adverse effects from CLA in any human studies and it is important to note that CLA is an essential fatty acid found naturally in products such as milk (Blankson, et al, ). The amount of fat from the CLA is about 5 % of the total dietary fat intake.
- Muscle biopsy. A muscle biopsy will be taken before and after the training from the *Vastus lateralis* (outer leg) muscle. Dr. Tarnopolsky has performed over 10,000 of these in patients and healthy control subjects ranging in age from 1 week to 90 y. with the following complications:
- 4/10,000 with a local skin infection.
- 6/10,000 with a fibrous lump at the site of biopsy (connective tissue); all disappeared with massage after < 1 week.
- 4/10,000 with a small patch of numbness just past the biopsy incision (size of a quarter) due to cutting a small sensory nerve branch. In all cases complete recovery occurred in < 3 months.
- The muscle usually has a dull ache for 24-48 h (markedly reduced with ice and mild analgesics such as Tylenol or Advil).
- In theory, one could damage a small motor branch of the m. *Vastus lateralis* and partially weaken the lower aspect of the muscle. This should not affect function for this muscle is only one of the four that have a role in knee extension. However, it has not been observed in any of the patients biopsied by Dr. Tarnopolsky.
- DEXA Scan: A DEXA scan is the most accurate measurement of body fat mass currently available. It uses a very low intensity radioactive beam to measure the amount of muscle and fat. The amount of radiation exposure is less than a cross-Canada airplane flight and much less than a standard chest X-ray (> 10 X less). There are no known risks with this test.
- Blood sampling. A single needle stick (Venipuncture) will be used to take blood three times during the study (pre-, mid- and post-test). The possibility of a small bruise at the site exists. The total amount of blood taken at any time is going to be 15 ml, which should have no negative effects.
- Muscle Testing/Training: Muscle strength testing may pose some risk for ligamentous or tendon injury. Knee extension strength is a standard testing and training method used in the elderly. In the last study that we completed in older adults (N = 28), there were no reported injuries from the testing or training. There are a number of these persons still participating in the “Seniors Exercise Program”.
- Exercise stress testing. We will not allow participants with abnormal resting ECGs or a blood pressure of > 160 mmHg systolic and/or 95 mm Hg diastolic to complete the exercise protocol (Bruce). It has been determined that the risk of a cardiac event during such testing is about 1/100,000. Subjects will feel tired at the end of the study and possibly light headed.

1. How will you manage and minimize the risks?

- Muscle Testing/Training: The training program that we have devised (see also Brose, et al.,  *J Gerontol A Biol Sci Med Sci.*, 58:B11-9, 2003), starts with low weights and gradually progresses as the individual adapts to the program. There is a light warm-up before each testing and training session. Any reports of tendon/ligament pain will be assessed by one of the supervising physicians and appropriate external consultation sought if it does not resolve with minor interventions.
- Serum CK activity, bilirubin, gamma-glutamyl transferase (GGT), and creatinine will be taken pre-, at 6 months, and at the end of the study. The pre- and + 6 month values will be provided to two local staff physicians (gastroenterologist and cardiologist) who will review the data and determine if there are any alterations that require further investigation or withdrawl from the study.
- Exercise stress testing. Prior to starting the study, each participant will have a symptom limited exercise stress test completed to determine maximal oxygen consumption and to determine if there are any ECG abnormalities. Any abnormalities that are deemed to be a risk to participation in an exercise program will be used as an exclusion criteria and the family doctor will be provided with the test results for appropriate action. Dr. C. Demers will review the pre-study and + 6 month tests for study exclusion or study withdrawal at the 6 month testing period. A defibrillator will be on hand at the time of the testing and each subject will have a 12 lead ECG.
- General health risk. Each participant will complete a health questionnaire and will be screened by the research coordinator to ensure that there are no contra-indications to participation. The family doctor of each potential candidate will be contacted to determine whether there are any contra-indications to participation. Specific exclusion criteria will include: known coronary artery disease, congestive heart failure, renal failure, diabetes requiring insulin or > 5 mg Glyburide or equivalent, renal failure (creatinine > 120, potassium > 5.00 mmol/L), previous stroke, uncontrolled hypertension or hypertension requiring more than mono-pharmacotherapy, inability to consent due to cognitive difficulties, chronic obstructive pulmonary disease (FVC or FEV1 < 85 % of age predicted mean value or requiring any medication other than a puffer as needed) or joint related problem that would exclude them from performing the strength training. Furthermore, we shall exclude persons who have a hip or lumber spine bone mineral density that is > 2 SD below the age adjusted mean values (based upon the DEXA scan).

1. (a) Will participants be reimbursed for expenses? [X] YES [ ] NO

If YES, please provide amount(s) and item(s) covered.

Participants will be reimbursed for their time commitment to the study for testing and follow-ups and for travel to and from the McMaster campus (or parking, if applicable). The estimated cost is about and this will be provided to each participant (extra will not be given to those randomized to exercise for this supervised training is inherently valuable (see below and above)).

(b) What other inducement or compensation is offered to participants?

The strength testing, diet assessment, VO2max testing, and body composition are all variables of interest that some people pay for at health clubs.

All participants will be given supervision on a daily stretching program before and again at 6 months.

The 50 % of people who will exercise will also benefit by the fact that they will have free, supervised exercise training. The 50 % who will not be exercising will be taught a stretching program and will come in to the center every 2 weeks to review this and complete the stretches as part of a group.

**SECTION D – PLAN FOR OBTAINING INFORMED CONSENT**

***Please complete the attached “Informed Consent Checklist” prior to completing this section.***

1. Describe how participants are to be recruited; if using advertisements include a sample with date.

Subjects will be recruited through advertisements around McMaster Campus, at local grocery stores and community centers and via a brief television appearance on the local Cable TV network in Hamilton (This was used successfully in an earlier training study in the elderly and more than twice the required subjects were recruited within a week).

1. Describe the relationship between the investigator(s) and the participant(s). Who will obtain consent?

Subjects will be not likely be known by any of the primary investigators. Informed consent will be obtained by either the study coordinator or one of the primary investigators.

1. Are participants competent to consent? [X ] YES [ ] NO If NO, describe the alternate source of consent.

If a minor, describe the procedure to be used. Where applicable, describe how you will obtain assent.

1. How will you assure ongoing consent from research participants?

The exercising participants will be required to come to the exercise training center twice a week (50 %). We will assume that their voluntary participation will indicate a willingness to continue in the study. There will be a mid-study (6 months) testing session where all aspects of the study will be reviewed. For the 50 % of participants who are not exercise training, they will come in for a 30 minute stretching session and to review any questions that arise.

1. What procedures will be followed for participants who wish to withdraw, either during or after the study?

Subjects who withdraw prior to randomization or within the first week (i.e., before the first testing session) will not be considered to have started the study and will be thanked for their interest. There are a few individuals who may use the study as a vehicle to obtain free exercise training and may drop out if they are not randomized to the exercise group. In our experience, this is about 5 – 10 % of all persons and we will notify people whether they are in the exercise groups or not before the first testing session. Once in the study (i.e., after the first testing session) all drop-outs will be considered in an intention to treat analysis of the data and the reason for the withdrawal will be noted and will be part of the final manuscript. Participants who experience any side effect from the study (i.e., tendonitis from exercise training) will be covered for the costs of treatment (such as physiotherapy or anti-inflammatories) and if they drop-out because of a negative consequence of the study, they will be given the full remuneration.

1. Will you be contacting participants either by telephone or letter during the project? [X] YES [ ] NO

If YES, append a copy of the telephone script or list of questions with the research protocol.

Participants will be contacted by telephone to book times for their testing sessions in order to fit into their schedule.

“Hello, this is ____________ calling from the older adult training study. We would like to plan the times for your testing sessions. We have a number of open testing sessions available including, ………… dates. If none of these times is convenient for you, are there times that would be more appropriate for you? Do you know how to get to the McMaster Medical Center, if not, we can send you a map or explain it over the phone. Thank you for participating in the study and we look forward to seeing you on (time and date).”

If participants miss more than one exercise session, they will be contacted to see if there is anything wrong (i.e., are they ill, injured, still interested in participating?).

“Hello, this is ____________ calling from the older adult training study. We noticed that you did not attend the exercise session on (date). I do hope that you and your family are healthy and hope to see you again soon. (if not home – “At your earliest convenience could you please give me a call at (number) if there are any questions.” Thank you again for participating in the study and we look forward to seeing you on (if they are home), or if they are not home …look forward to hearing from you.”

**SECTION E – STEPS TO BE TAKEN TO ENSURE CONFIDENTIALITY OF DATA**

1. How will identity of participant(s) be protected?

The names of the participants will be used only on the original documents for identification, remunerations cheque mailing, and to send a copy of the final paper and a lay summary with their own personal data. The publications will not contain any identifiers that could possibly be used to identify the person’s identity.

1. Where and how will the data be stored; and who will supervise access to the data to ensure confidentiality is maintained?

Dr. Tarnopolsky will ultimately supervise access to the data in Hamilton and Dr. Doherty in London. The research coordinator (McMaster site) will organize and centralize the data and will store a “mega copy” of all the data at McMaster in his or her office (4U20) – locked office and filing cabinet.
